# Prevalence of Parkinson’s disease among adults aged 45 years and older in China: a cross-sectional study based on the China health and retirement longitudinal study

**DOI:** 10.1186/s12889-024-18653-0

**Published:** 2024-05-02

**Authors:** Detao Meng, Jiayu Wu, Xinyu Huang, Xiaoxiao Liang, Boyan Fang

**Affiliations:** 1 Parkinson Medical Center, Beijing Rehabilitation Hospital, Capital Medical University, Shijingshan District, BadachuBejing, 100144 Xixiazhuang China; 2 Department of Neurorehabilitation, Xiamen Humanity Rehabilitation Hospital, Fujian, China

**Keywords:** Parkinson’s disease, Prevalence, Chinese mainland population, Aging

## Abstract

**Background:**

In recent decades, China has experienced a rapid increase in the number of elderly individuals and life expectancy, as well as industrialization, which is associated with an increased prevalence of Parkinson's disease (PD). However, inconsistent results have recently been reported. Therefore, this study aimed to investigate the prevalence and distribution characteristics of PD among individuals aged 45 years and older.

**Methods:**

Using data from the China Health and Retirement Longitudinal Study (CHARLS), we attempted to estimate the prevalence of PD and its distribution characteristics among 19,034 individuals aged 45 years and older residing in 446 communities/villages within 27 provinces/autonomous regions/municipalities in mainland China. Cases were established based on a doctor's previous diagnosis. Crude and age-adjusted prevalence rates were calculated and stratified by age, sex, area of residence, education level, marital status, and geographic region. Logistic regression models were used to identify risk factors associated with PD.

**Results:**

We identified 178 patients with PD among 19,034 residents aged 45 years and older. The crude prevalence was 0.94%, and the age-adjusted prevalence was 0.82% for individuals aged 45 years and older. The prevalence of PD increased with age (*P* < 0.001). No significant differences were found in terms of sex, area of residence, or education level. Stratified by geographic region, the prevalence of PD was greater in North and Northwest China and lower in southern China (*p* < 0.001). Multiple regression analyses showed that age was a significant risk factor for PD.

**Conclusion:**

The prevalence of PD increased with age in the Chinese population.

## Introduction

Over the past four decades, China has experienced an increase in both the number and proportion of elderly individuals, accompanied by an extended lifespan. According to the data of the China Statistical Yearbook (22) [1], there were only 49 million adults aged 65 years and above in China, accounting for 4.9% of the population, in 1982. However, by 2021, this number increased to 201 million, making up 14.2% of the population [1]. In 1981, the life expectancy was 67.77 years, but by 2020, the life expectancy had increased to 77.93 years [1]. Currently, China has the largest population of older people worldwide [2]. Simultaneously, China underwent unparalleled industrialization during this period [3, 4]. All of these factors, including an aging population, increased longevity, and environmental pollution caused by rapid industrialization, contribute to the increasing prevalence of Parkinson’s disease (PD) [5–9]. Consequently, according to the Global Burden of Diseases, Injuries, and Risk Factors Study (GBD) 2016, adjusted prevalence rates of PD in China have exhibited a more pronounced increase than those in any other country globally, doubling from 1990 to 2016 [6, 7]. However, inconsistent findings have emerged from two studies conducted on this subject matter [4, 10]. One nationwide study involving 24,117 participants aged > 60 years reported that the prevalence of PD was estimated to be 1.37% (95% confidence interval (CI): 1.02%-1.73%), with no significant change observed previously in terms of the PD population prevalence percentage [4]. Another study indicated an overall PD prevalence rate among individuals aged 65 years and above in China of approximately 1.86%, suggesting a consistent PD prevalence over time within the Chinese population segment mentioned above. Therefore, whether there has been a rapid increase in the prevalence of PD is still controversial. While PD is an age-related disease, the misconception that it only affects older people should be dispelled [5]. Nearly 25% of affected individuals are younger than 65 years of age, and 5–10% are younger than 50 years of age [5]. Most recent studies on PD prevalence in China primarily focused on the elderly population, leaving a dearth of data for middle-aged individuals [4, 10]. These patients have an early onset and a relatively long course of disease. Understanding the prevalence of these patients is also essential to effectively manage the disease.

Age, sex, and rural residence are regarded as risk factors for PD [11, 12]. However, evidence on this association remains limited and inconsistent. Recent studies have reported some contrasting results [4, 10]. In addition, education level, marital status, and geographical region may impact the prevalence of PD in the mainland Chinese population. Due to inconsistent findings in previous studies and the limited availability of nationwide epidemiological data on PD in China, we conducted this study to explore the prevalence and distribution characteristics of PD among individuals aged 45 years and above in the mainland population using data from the China Health and Retirement Longitudinal Study (CHARLS) to provide a reference for the prevention and management of PD in China.

## Materials and methods

### Data sources and study population

**Fig. 1:**
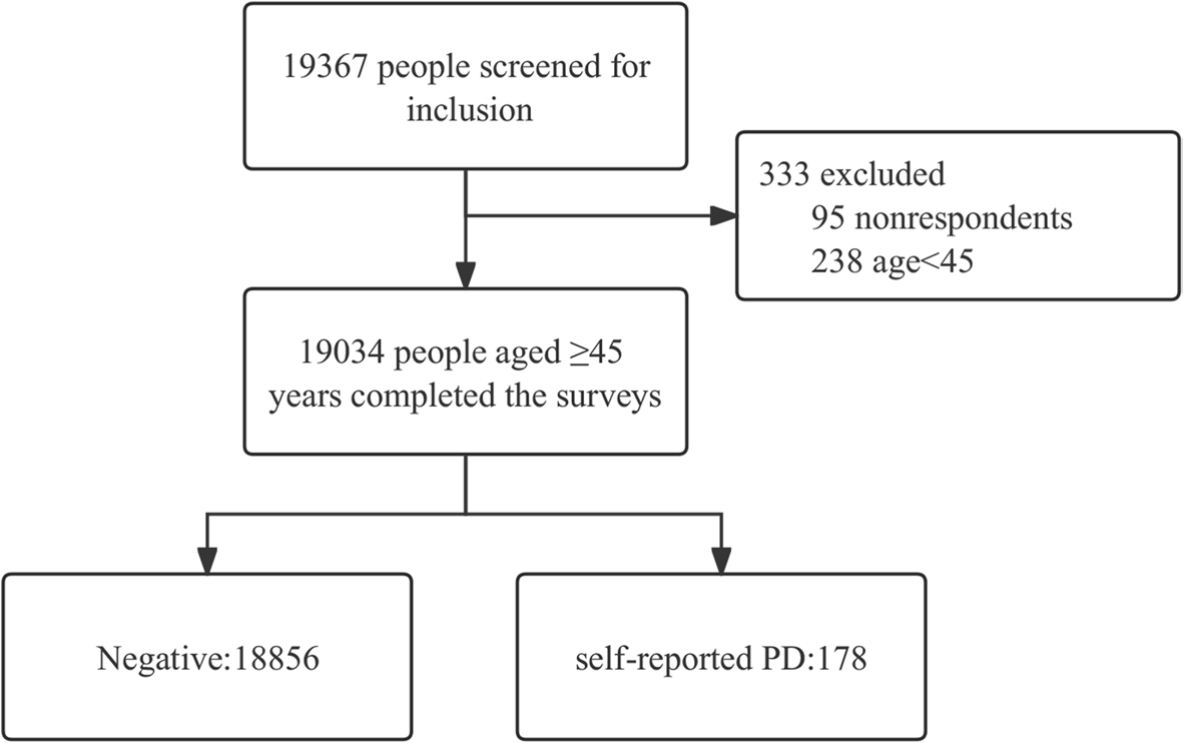
The study flowchart. PD: Parkinson’s disease

The data utilized in this study were acquired from the China Health and Retirement Longitudinal Study [13] (CHARLS) database. CHARLS is an epidemiological survey project initiated by the National Development Research Institute of Peking University in 2008. Its primary objective is to analyze the aging population in China and promote interdisciplinary research. CHARLS is an open study, and the data can be accessed through the website https://charls.charlsdata.com/users/sign_in/zh-cn.html. Five nationwide follow-up CHARLS surveys were conducted in 2011, 2013, 2015, 2018, and most recently in 2020, providing the dataset employed for this investigation. Our study was approved by the ethics committee of the Beijing Rehabilitation Hospital. The methods used in this study adhered to relevant guidelines and regulations. Before participating in the survey, all participants signed an informed consent form approved by the ethics committee of Peking University. Given the illiteracy of some participants, the investigator obtained informed consent by presenting and explaining its contents to them. Participants then provide their informed consent through handprints. The general study design and flowchart are depicted in Fig. 1.

The inclusion criteria for the present study were as follows: (1) aged 45 years and above and (2) provided informed consent before participation. The exclusion criteria were as follows: (1) under 45 years of age and (2) no answer to the question “Have you ever been diagnosed with PD by a physician?”.

### Sampling method

The data in this study were obtained using a multistage probability sampling methodology. Detailed descriptions of the sampling methodology have been provided in a previous paper [13]. First, 150 county-level units were selected at random from a sampling frame of all county-level units except Tibet, Taiwan, Hong Kong, Macao, Ningxia, and Hainan using the probability proportional to size (PPS) sampling technique. The sample was stratified by region and within regions by urban or rural areas and gross domestic product (GDP). The lowest level of government organization was used as the primary sampling unit (PSU), which included administrative villages in rural areas and neighborhoods in urban areas. PSUs were selected in each county-level unit by the PPS sampling method. A total of 450 communities/villages spanning 150 counties within 28 provinces/autonomous regions/municipalities were selected. However, in the fifth follow-up survey, data for 4 communities were lacking; thus, our survey covered 446 communities/villages within 27 provinces/autonomous regions.

### Diagnosis of PD

The diagnosis of PD was established based on a doctor's previous diagnosis. The participants were queried about whether they had received a formal PD diagnosis; if they had, then the participant was considered to have PD, and if they had not, then they were deemed not to have PD.

### Statistical analysis

The 2020 CHARLS cross-sectional survey data were cleaned and merged using Stata/MP 17.0 statistical software, and the statistical analysis was performed using SPSS 21.0. All calculations were weighted to represent the general adult population aged 45 years and above in China according to the 2020 population census. Crude prevalence rates and age-adjusted rates were calculated by direct standardization to the 2020 China population census [14]. For the estimates, 95% CIs were established. Descriptive statistics were employed to evaluate the demographic information of the participants. Continuous variables are presented as the means and standard deviations, while categorical variables are represented by frequencies and percentages. Categorical variables were compared using the chi-squared test. A logistic regression model was used to identify the main risk factors for PD, including sex; age group (45–49, 50–54, 55–59, 60–64, 65–69, 70–74, 75–80, and ≥ 80 years); area of residence (rural vs. urban); education level (illiterate vs. primary school vs. junior high school and above); and marital status (widowed, divorced or living alone, or married), by entering these variables using forward stepwise methods. The seven geographical regions were classified based on their geographical characteristics [15]: East China, North China, Northeast China, Northwest China, South Central China, Central China, and Southwest China. The geographic region was also included as a risk factor in logistic regression analysis. A significance level of *p* < 0.05 was used to determine statistical significance. The results are presented as 95% CIs.

## Results

Nineteen thousand three hundred sixty-seven adults were invited to participate in the survey, of whom 333 were excluded (Fig. 1). A total of 19,034 adults aged 45 years and above completed the survey; 10,019 were female (52.6%), and 9,015 were male (47.4%), with an average age of 61.8 ± 9.8 years. The demographic data are shown in Table 1.

**Table 1:** Demographics of the study population

	*N* (%)	Female	Male
Gender	…	52.60%	47.40%
Age, years			
45-49	1833 (9.6%)	1079 (10.8%)	754 (8.4%)
50-54	3462 (18.2%)	1830 (18.3%)	1632 (18.1%)
55-59	3166 (16.6%)	1662 (16.6%)	1504 (16.7%)
60-64	3313 (17.4%)	1673 (16.7%)	1640 (18.2%)
65-69	3158 (16.6%)	1657 (16.5%)	1501 (16.7%)
70-74	1928 (10.1%)	958 (9.6%)	970 (10.8%)
75-80	1191 (6.3%)	620 (6.2%)	571 (6.3%)
>80	983 (5.2%)	540 (5.4%)	443 (4.9%)
Residence			
Urban	6943 (36.5%)	3662 (36.6%)	3281 (36.4%)
Rural	12082 (63.5%)	6353 (63.4%)	5729 (63.6%)
Education level			
Illiterate	4220 (22.2%)	3377 (33.7%)	843 (9.4%)
Primary School	8152 (42.8%)	4068 (40.6%)	4084 (45.3%)
Junior high school and above	6662 (35.0%)	2574 (25.7%)	4088 (45.3%)
Marital status			
Married	15941 (83.8%)	7907 (78.9%)	8034 (89.1%)
Widowed	2624 (13.8%)	1946 (19.4%)	678 (7.5%)
Divorced or living alone	469 (2.5%)	166 (1.7%)	303 (3.4%)
National region			
Northeast China	1179 (6.2%)	637 (6.36%)	542 (6.01%)
North China	2401 (12.6%)	1241 (12.39%)	1160 (12.87%)
East China	5988 (31.5%)	3156 (31.50%)	2832 (31.41%)
South China	1636 (8.6%)	872 (8.70%)	764 (8.47%)
Central China	3035 (15.9%)	1590 (15.87%)	1445 (16.03%)
Northwest China	1383 (7.3%)	731 (7.30%)	652 (7.23%)
Southwest China	3412 (17.9%)	1792 (17.89%)	1620 (17.97%)

**Fig. 2:**
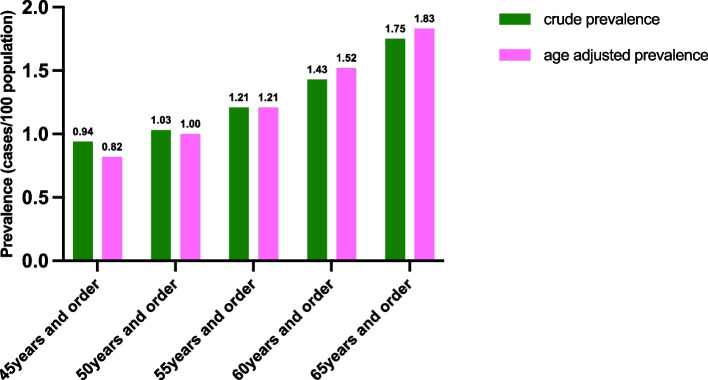
The crude prevalence and age adjusted prevalence of Parkinson’s disease

A total of 178 adults aged 45 years and above were reported to have PD; the crude prevalence rate was 0.94%, and the age-adjusted prevalence rate was 0.82% according to the 2020 population census. In addition, we estimated the crude and age-adjusted prevalence rates of PD among people aged 50 years and above, 55 years and above, 60 years and above, and 65 years and above. The crude and age-adjusted prevalence rates of PD are shown in Fig. 2. The crude prevalence rate was 0.32% among 8461 middle-aged individuals aged 45–59 years as showed in Table 1. The prevalence of PD is significantly higher in the elderly population compared to middle-aged individuals (*p* < 0.001).

**Fig. 3:**
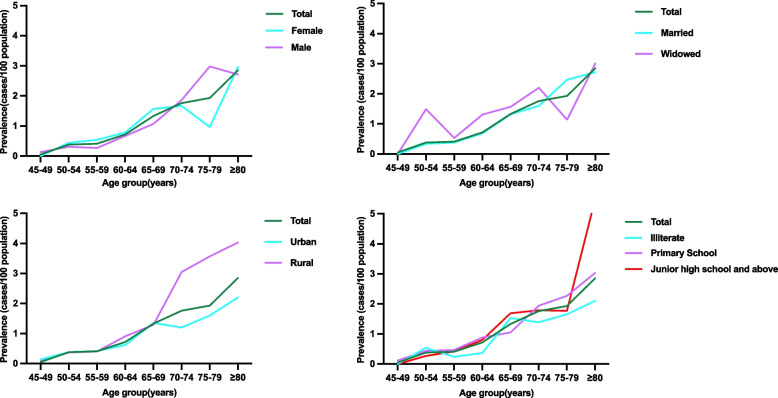
Age trends in the prevalence of Parkinson’s Disease stratified by gender, residence, marital status, and education levels

Stratified by age group, the prevalence rates of PD significantly increased with age (*p* < 0.001), from 0.05% among 1833 individuals aged 45–49 years to 2.85% among 985 individuals aged > 80 years (Table 2). The male and female populations had PD prevalence rates of 0.94% and 0.93%, respectively. The prevalence rates of PD in urban and rural areas were 1.11% and 0.84%, respectively. Regarding education level, the prevalence rates of PD were 1.09%, 0.99%, and 0.77% among people with illiteracy, a primary school education, and a junior high school or above education, respectively. In terms of marital status, the prevalence rates of PD were 0.80%, 1.79%, and 0.64% for married, widowed, and divorced people or those living alone, respectively (Table 2). No significant differences were found in terms of sex, area of residence, or education level. The prevalence of PD was significantly higher among widowed individuals than among married or divorced individuals or those living alone (*p* < 0.001). Age trends in the prevalence of PD stratified by sex, area of residence, education level, and marital status are shown in Fig. 3. Stratified by geographic region, the prevalence of PD was greater in North and Northwest China and lower in southern China (*p* < 0.001) (Table 2). Table 3 shows the results of the multilevel logistic regression analysis. Age was identified as an independent risk factor for PD.

**Table 2:** Stratified estimates of PD prevalence based on sociodemographic factors

	No. of no PD Cases	No. of PD Cases	PD Prevalence (%)	95%CI	*P*-value
Age, year					<0.001
45-49	1832	1	0.05	（-0.10-0.20）	
50-54	3449	13	0.38	（0.20-0.60）	
55-59	3153	13	0.41	（0.20-0.60）	
60-64	3289	24	0.72	（0.40-1.00）	
65-69	3116	42	1.33	（0.90-1.70）	
70-74	1894	34	1.76	（0.12-0.24）	
75-80	1168	23	1.93	（0.11-0.27）	
>80	955	28	2.85	（0.18-0.39）	
>45	18856	178	0.94	（0.80-1.10）	
>50	17024	177	1.03	（0.90-1.20）	
>55	13575	164	1.21	（1.00-1.40）	
>60	10422	151	1.43	（1.20-1.60）	
>65	7133	127	1.75	（1.40-2.00）	
45-59	8461	27	0.32	(0.20-0.40)	
Gender					0.963
Female	9925	94	0.94	（0.70-1.10）	
Male	8931	84	0.93	（0.70-1.10）	
Residence					0.06
Urban	6866	77	1.11	（0.90-1.40）	
Rural	11981	101	0.84	（0.70-1.00）	
Education level					0.177
Illiterate	4174	46	1.09	（0.80-1.40）	
Primary School	8071	81	0.99	（0.80-1.20）	
Junior high school and above	6611	51	0.77	（0.60-1.00）	
Marital status					<0.001
Married	15813	128	0.80	（0.70-0.90）	
Widowed	2577	47	1.79	（1.30-2.30）	
Divorced or living alone	466	3	0.64	（0.10-1.40）	
National region					0.025
Northeast China	1179	13	1.10	(0.50-1.70)	
North China	2401	34	1.42	(0.90-1.90)	
East China	5988	47	0.78	(0.60-1.00)	
South China	1636	11	0.67	(0.30-1.10)	
Central China	3035	28	0.92	(0.60-1.30)	
Northwest China	1383	20	1.45	(0.80-2.10)	
Southwest China	3412	25	0.73	(0.40-1.00)	

*PD* Parkinson’s disease, *CI* confidence interval

**Table 3:** Multivariate analysis for independent predictors of PD

PD	OR(95% CI)	*P* value
Age, year		
45–49	1.000	
50–54	6.846 (0.895, 52.392)	0.064
55–59	7.607 (0.993, 58.246)	0.051
60–64	13.765 (1.858, 101.991)	0.010
65–69	25.48 (3.491, 185.956)	0.001
70–74	33.899 (4.614, 249.07)	0.001
75–80	37.428 (5.008, 279.731)	< 0.0001
> 80	55.138 (7.349, 413.707)	< 0.0001
Gender		
Female	1.000	
Male	0.974 (0.703, 1.35)	0.875
Residence		
Urban	1.000	
Rural	0.734 (0.533, 1.01)	0.058
Education level		
Illiterate	1.000	
Primary School	1.197 (0.806, 1.779)	0.373
Junior high school and above	1.067 (0.662, 1.719)	0.790
Marital status		
Married	1.000	
Widowed	1.155 (0.787, 1.695)	0.462
Divorced or living alone	0.809 (0.255, 2.564)	0.718
National region		
Northeast China	1.000	
North China	1.397 (0.732, 2.669)	0.311
East China	0.717 (0.383, 1.345)	0.300
South China	0.589 (0.261, 1.328)	0.202
Central China	0.898 (0.460, 1.751)	0.751
Northwest China	1.504 (0.738, 3.068)	0.262
Southwest China	0.667 (0.335, 1.328)	0.250

*PD* Parkinson’s disease, *OR* odds ratio, *CI* confidence interval

## Discussion

In this study, we investigated the prevalence of PD and its distribution among individuals aged 45 years and older of different ages, sexes, areas of residence, education levels, and geographic regions in China. To our knowledge, our study is the largest in the past 30 years, covering 27 provinces/autonomous regions/municipalities. The age-adjusted PD prevalence rates were 0.82% in the middle-aged and elderly population aged 45 years and above and 1.52% in the elderly population aged 60 years and above. There is a significant increase in the prevalence of PD among the elderly compared to the middle-aged population. Age was identified as an independent risk factor for PD. A significant increase in PD prevalence was observed with age, with the highest prevalence rates observed among those aged > 80 years. Moreover, our study revealed that there were no significant differences in PD prevalence in terms of sex, area of residence, education level, marital status, or geographic region.

Previous studies have reported the prevalence of PD in the Chinese mainland population, but variations in age definitions and historical periods have led to differences in these estimates. Wang et al. [16] conducted an epidemiological survey in 1986, reporting a PD prevalence rate of 1.14% among individuals aged 60 and above across 117 areas in 29 provinces, municipalities, and autonomous regions. Zhang et al. [17] investigated 58 communities in 79 villages in Beijing, Xi'an, and Shanghai from 1997 to 1998, reporting PD prevalence rates of 1.07% and 1.7% among individuals over the ages of 55 and 65, respectively. Qi et al. [4] surveyed six provinces, namely, Beijing, Shanghai, Hubei, Sichuan, Guangxi, and Yunnan, and reported a PD prevalence rate of 1.37% among individuals older than 60 years in 2015. Song et al. [10] conducted an epidemiological survey among residents of 11 provincial capitals or municipalities and 10 rural counties in China in 2019 and reported a prevalence of PD of 1.86% among individuals aged 65 years and above. We found a PD prevalence of 1.52% among individuals aged 60 years and above, which is slightly greater than that reported by Wang et al. [16] 30 years ago, and a PD prevalence of 1.83% among individuals aged 65 years and above, which is also slightly greater than that reported by Zhang et al. [17] 20 years ago. Therefore, our results support that the prevalence of PD has increased with the aging of the Chinese population. As in China, some high-income countries, including those in North America and Western Europe, are also experiencing an increase in PD prevalence because of aging populations, longer disease durations, and potential changes in environmental or societal risk factors [18–21]. Over the past three decades, the prevalence of PD in the Middle East and North Africa region has increased as well [22]. Some East Asian countries, such as Korea and Japan, also have rapidly aging populations [23], and the prevalence of PD has also increased [24, 25]. Globally, increased overall age-standardized incidence rates, prevalence rates, and years lived with disability due to PD were also reported in most regions and countries from 1990 to 2019 [26]. Compared to 20 years ago, the number of people suffering from PD in China has increased substantially. Approximately 1.7 million individuals suffered from PD in 1997–1998 [17, 27]. However, for the past two decades, the total number of patients with PD in China has increased to 3.62 million in 2020 [4]. The burden of PD in China is much greater than that in other countries [6, 7] and may increase further in the coming decades with the aging population. Due to the increasing burden, PD necessitates an urgent public health response in China [28].

PD is a neurodegenerative disorder that primarily affects the elderly population. Previous studies have demonstrated an age-related increase in PD prevalence [10, 29–31]. Consistent with the findings of previous studies, our study revealed that older age was a risk factor for PD [29, 32]. The prevalence of PD increased significantly with age, and individuals aged > 80 years had the highest prevalence of PD, which is consistent with the findings of Song et al. [10]. Our study suggested that the prevalence of PD increases with age, which may be related to dopamine neuron degeneration in people with PD [32, 33]. Despite abundant evidence supporting aging as a pivotal risk factor for PD, its biological underpinnings remain elusive [33, 34]. Collier, T. J. et al. proposed that aging and PD coexist on a shared continuum that includes impaired proteasome/lysosome function, oxidative/nitrative damage, and increased inflammation and that neurons are particularly susceptible to mitochondrial dysfunction [34]. This shared biology suggests that aging actively creates a vulnerable preparkinsonian state [32, 34, 35]. The cellular mechanisms underlying dopamine neuron death during normal aging are accelerated or exaggerated in individuals with a genetic predisposition or exposure to environmental factors associated with PD [32].

Previous studies have suggested that sex [36, 37], area of residence [10, 38, 39], and education level [40–42] can influence the prevalence of PD. Specifically, a higher PD prevalence has been observed among males [36, 37], urban residents [39], and people with higher education levels [40–42]. However, some studies have reported conflicting findings, suggesting that factors such as sex [4, 30, 43, 44], area of residence [10], and education [45] may not have or may have opposite impacts on the prevalence of PD. Our study supports the findings of previous research [31, 44, 46, 47], indicating that there are no significant associations between sex, area of residence or education level and PD prevalence. Although we obtained a negative result, the effects of sex, area of residence, and education level on PD prevalence are still inconclusive, and further larger-scale studies are needed to confirm these results. Stratified by marital status, the prevalence of PD was greater in the widowed group than in the married group; however, this difference was observed only in univariate analysis and was not tested in multivariate analysis. This can likely be explained by the older age of the widowed group. Geographical differences were also evaluated in our study. The prevalence of PD was greater in North and Northwest China and lower in southern China according to univariate analysis, but no significant difference was found via multivariate regression analysis. Reports on this topic are rather scarce. However, additional studies with larger sample sizes are needed to confirm these conclusions.

This study has certain limitations. First, the PD prevalence results of this study were based on a doctor's previous diagnosis. Disparities in healthcare resources [48] may have led to an overestimation or underestimation of PD prevalence. Second, as a cross-sectional study, this research captured the prevalence of PD solely at a specific moment and failed to elucidate its dynamic changes and developmental trends. Future research should focus on refining the study design and improving the data acquisition methods to enhance the reliability and accuracy of the study findings. Moreover, our study primarily focused on the prevalence and distribution characteristics of PD; therefore, only sociodemographic variables were considered. However, it is important to note that other factors such as genetic predisposition, lifestyle habits, and environmental influences have also been linked to the development of PD [5, 49, 50].The establishment of a comprehensive understanding of the relationship between these factors and PD necessitates further analysis, in order to develop effective prevention policies in China.

## Conclusions

The findings of this study suggest that the prevalence of PD is increasing in China due to its aging population. As populations age and medical facilities improve, the prevalence of PD will likely continue to rise, resulting in considerable health, social, and economic impacts. Therefore, raising public awareness of the disease and implementing effective measures to prevent or treat PD are crucial, as PD has become a major public health issue that cannot be ignored.

## Data Availability

The data used in this study are released data by CHARLS for public use. Permissions were acquired to access the data used in our research, which were granted by CHARLS team. The raw data is available on website (https://charls.pku.edu.cn/en).

## Abbreviations

PD: Parkinson’s disease
CHARLS: China Health and Retirement Longitudinal Study
CI: Confidence interval
